# What Happens in Midlife Doesn’t Stay in Midlife: Exploring Longitudinal Pathways to Health in MIDUS

**DOI:** 10.1093/geroni/igaf122.1538

**Published:** 2025-12-31

**Authors:** Margie Lachman

**Affiliations:** Brandeis University, Waltham, Massachusetts, United States

## Abstract

There is growing evidence that physical and psychosocial factors in midlife play an important role in later life functioning. Blood pressure, lung function, glucose levels, cholesterol, sleep, and weight in midlife are strong predictors of health and cognition in later life. Psychosocial factors such as the sense of control, purpose in life, and social support in midlife also are important as they can motivate health-promoting behaviors that contribute to maintaining functional and cognitive health and reducing chronic conditions in later life. The relationships between midlife functioning and later life health and cognition are moderated by educational attainment and mediated by biomarkers including inflammation and allostatic load. With the rich Midlife in the United States (MIDUS) data we can examine the long-term cumulative effects and pathways to health over 30 years, showing differential trajectories in the transition from midlife into older ages. Health in midlife provides a window on later life and can inform preventative interventions to optimize functioning and health span. There is much interest in identifying modifiable lifestyle factors that can promote healthy aging. Results from multiple studies using the MIDUS dataset provide convincing evidence that addressing risk factors in midlife and adopting adaptive behaviors such as engaging in physical and cognitive activity can have long-term benefits for health outcomes and longevity. The discussion will focus on middle age as an ideal time to make lifestyle changes to increase health span throughout later life.

